# Three-dimensional Analysis of the Distribution of Smooth and Skeletal Muscle Tissue Around the Female Urethra

**DOI:** 10.1007/s00192-025-06045-w

**Published:** 2025-01-20

**Authors:** Tong Liu, Satoru Muro, Suthasinee Tharnmanularp, Keiichi Akita

**Affiliations:** 1 Department of Clinical Anatomy, Tokyo Medical and Dental University (TMDU), 1-5-45 Yushima, Bunkyo-Ku, Tokyo, 113-8510 Japan; 2 Princess Srisavangavadhana College of Medicine, Chulabhorn Royal Academy, Bangkok, Thailand

**Keywords:** External urethral sphincter, Female urethral function, Levator ani, Urinary incontinence

## Abstract

**Introduction and Hypothesis:**

The urethra is surrounded by layers of smooth muscle, including inner longitudinal and outer circler muscles, as well as the skeletal muscle of the external urethral sphincter. However, the extent of these muscles and their relationship with the levator ani (LA) remain unclear. This study aimed to clarify the composition of muscle layers around the female urethra and their three-dimensional arrangements.

**Methods:**

Pelvises from five female cadavers were collected for histological analysis. The surrounding urethral tissue was serially sectioned and stained with Elastica van Gieson stain to identify the connective and muscular tissues. Immunohistological staining with an anti-smooth muscle antibody was performed to confirm the distribution of the smooth and skeletal muscles. Thereafter, muscle layers were three-dimensionally reconstructed on the basis of serial histological sections.

**Results:**

The female urethra was surrounded by four muscle layers arranged from inner to outer layers in the following order: longitudinal smooth muscle, circular smooth muscle, the external urethral sphincter (EUS), and an outermost smooth muscle layer. These muscles surrounded the urethra and partially extended anteriorly. Moreover, smooth muscle fibers extending from the vagina were found between the LA and EUS.

**Conclusions:**

This study clarified the distribution of muscle tissue surrounding the female urethra, showing that the EUS is connected to the LA through the outermost smooth muscle layer. Thus, urination control likely involves both the urethral muscle layers and the pelvic floor muscles.

## Introduction

Recently, urinary incontinence in women caused by pelvic floor muscle injuries or other diseases has gained attention [1]. Understanding the muscular anatomy, including the distribution of muscles surrounding the urethra, is essential to fully understand the mechanisms of female urination and the pathological causes of urinary incontinence.

According to anatomical texts and several studies, the female urethra is surrounded by two smooth muscle layers: an inner longitudinal layer and an outer circular layer [2–5]. However, some studies suggest dividing the smooth muscle into three layers: the inner longitudinal layer, the outer circular layer, and the outermost longitudinal muscle layer [6, 7]. Outside the smooth muscle layers is a horseshoe-shaped skeletal muscle that encircles the urethra, commonly referred to as the external urethral sphincter (EUS) [8–10]. Research on the relationship between the peri-urethral and pelvic floor muscles in female individuals suggests that the inferior part of the EUS is firmly attached to the levator ani (LA) by a tendon in female fetuses [11].

Previous anatomical studies by Muro et al. showed that the pelvic floor consists of widespread skeletal and smooth muscles, suggesting that their interactions create its function [12–14]. In male individuals, the urethra is located immediately anterior to the rectum, and both the urethra and the anorectal canal are connected by skeletal and smooth muscles, suggesting a functional relationship [15, 16]. In female individuals, the urethra is located anterior to the vagina. Kato et al. reported that the smooth muscle surrounding the female urethra is more extensive than previously thought and linked to the smooth muscle surrounding the vagina. Moreover, the LA and smooth muscle fibers were in direct contact, playing an important role around the urethra as a mediator between the pubis, urethra, and LA. This structure suggests that the urethra is supported by the vagina, LA, and pubic bone [4]. However, the extent of these smooth and skeletal muscles and the impact of muscle distribution at different urethral levels on urination remain unclear.

This study aimed to clarify the layered structure of smooth and skeletal muscles distributed around the female urethra, particularly in relation to the LA muscle. Through this research, we hope to contribute to the clinical treatment of urinary incontinence, focusing on developing surgical techniques.

## Materials and Methods

The five female cadavers used in this study were provided by our department, following the “Act on Body Donation for Medical and Dental Education.” Each donor voluntarily agreed to donate their body parts for research and teaching before death after being informed about the procedures. We informed the bereaved family of the deceased’s consent after their passing and ensured no conflicts of interest existed. We displayed posters about the study and shared its results. All cadavers underwent arterial perfusion with 8% formalin and were preserved in a 30% alcohol solution. Donors with a history of pelvic anomalies were excluded from the study. All procedures complied with relevant standards and laws.

### Histology

For histological analysis, all pelvises from donors (ages at death 81, 79, 75, 84, and 75 years) were used. Two peri-urethral and three lateral urethral specimens were obtained. Among them, one peri-urethral specimen, approximately 8 cm thick, and one lateral urethral specimen, approximately 3.5 cm thick, were selected for histological observation through wide-range serial sectioning [17]. These two blocks were fixed in 10% formalin, dehydrated, embedded in paraffin, and sectioned into 5-μm thick sections at 1 mm intervals.

Elastica van Gieson staining was used to identify connective and muscular tissues in the histological sections. Immunostaining with anti-smooth actin (Ab-5694) was performed to confirm the distribution of smooth and skeletal muscle fibers. The specific steps have been previously described [4, 15, 17–20].

### Three-dimensional Reconstruction

Using the SrfII software (ver. R.11.00.00.0-H, Ratoc, Tokyo, Japan; http://www.ratoc.com/eng/index.html) to get the three-dimensional reconstruction, the female urethra and surrounding muscles were examined. Serial transverse sections from the two histologically examined blocks (from donors aged 81 and 79) were used for three-dimensional reconstructions. For the peri-urethral block, 37 slides covering the entire length of the urethra were chosen, and the EUS, smooth muscle, longitudinal smooth muscle, and circular smooth muscle surrounding the urethra were manually segmented after scanning all 78 serial sections. For the lateral urethral block, the structures (EUS, ligament, LA, pubic bone, smooth muscle, longitudinal and circular smooth muscles surrounding the urethra, urethra, vagina, and smooth muscle from the vaginal wall) were manually segmented after scanning all 35 serial sections. This technique was similar to that used in previous reports [17].

### Ethical Approval

The study was approved by the Ethics Committee of our Institute (approval number: M2018-006). All methods were performed in accordance with the relevant guidelines and regulations.

## Results

### Distribution of Muscle Tissue Around the Female Urethra

**Fig. 1:**
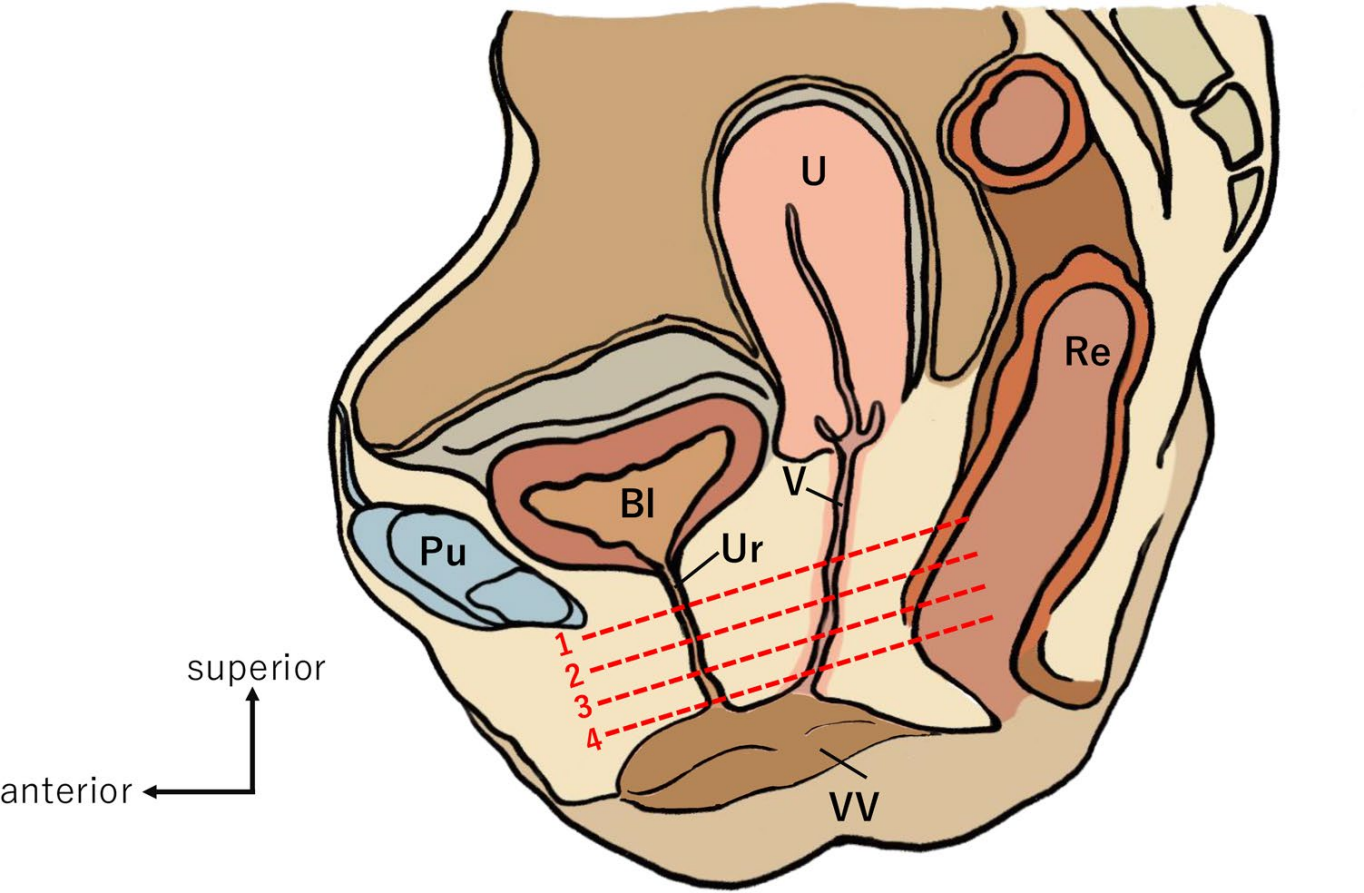
Illustration of female pelvic halves in the median plane (right side). Lines 1–4 indicate the level at which representative slides were selected. *Bl* bladder, *Pu* pubic bone, *Re* rectum, *U* uterus, *Ur* urethra, *V* vagina, *VV* vaginal vestibule

**Fig. 2:**
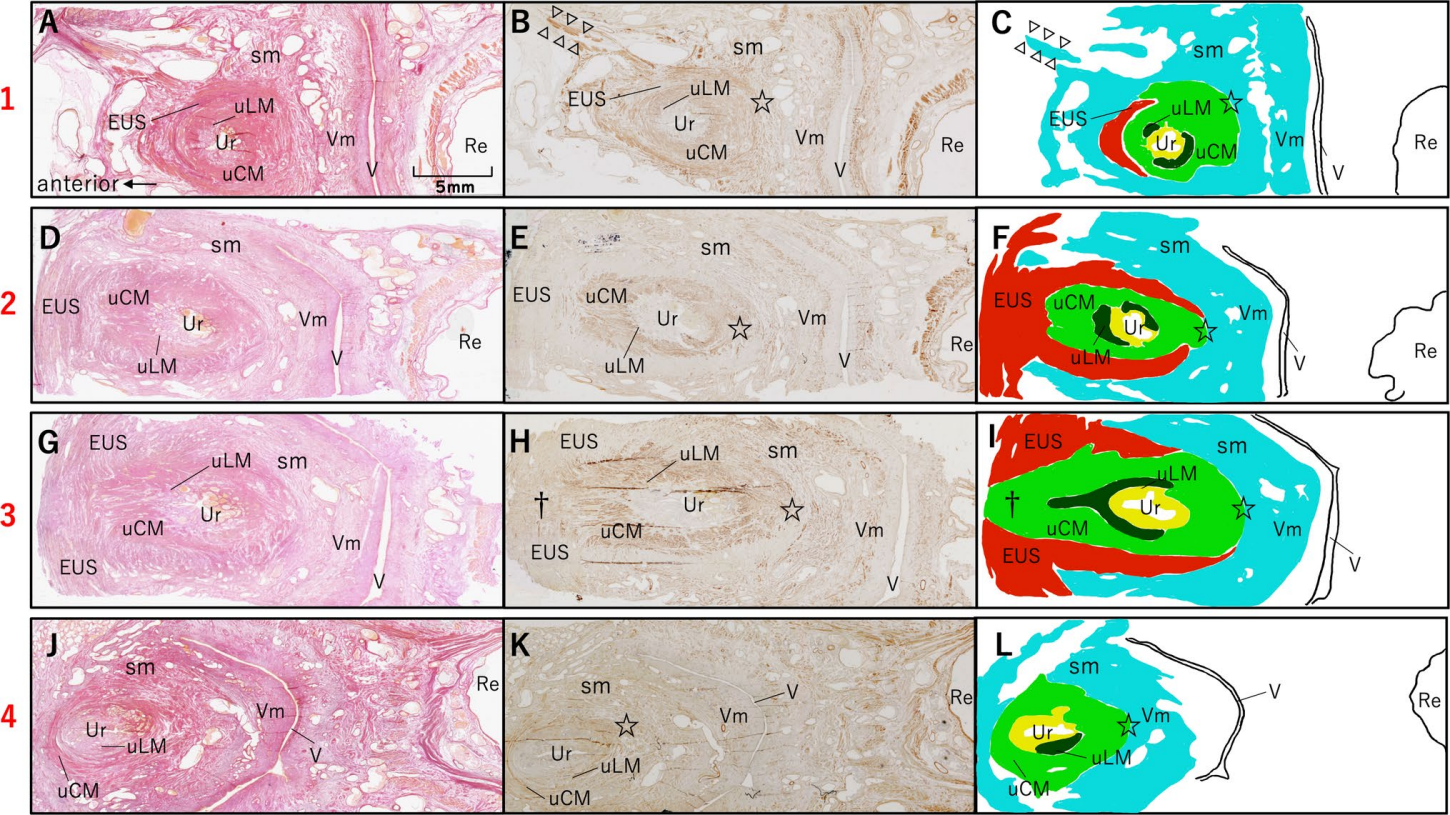
Histology of the smooth and skeletal muscular tissue around the urethra. Transverse histological sections of the smooth and skeletal muscles around the female urethra were obtained using EVG staining (a, d, g, and j) and immunostaining with an anti-smooth muscle antibody (b, e, h, and k). **a**–**c** Section of the proximal part of the female urethra located 11 mm from the urethral–vesical junction. The muscle tissue was arranged in the order of the uLM, uCM, and skeletal muscle from the inner to the outer layers, identified as the EUS. The outermost smooth muscle tissue protruded anterolaterally and extended the muscle fibers toward the pubic bone. **d**–**f** Section 7 mm from the inferior surface of a–c, in which the skeletal muscle surrounded the urethra in a horseshoe shape, lacked muscle fibers posteriorly. Posteriorly, in the urethra, the uCM and outermost smooth layers continued with each other (e, star). **g**–**i** A section 3 mm from the inferior surface of d–f. In the section at the distal part of the urethra, uLM and uCM extended anteriorly to separate the skeletal muscle layers (h, i, and dagger). **j**–**l** A section 7 mm from the inferior surface of g–i where no skeletal muscle was observed. *EUS,* external urethral sphincter; *Re,* rectum; *sm,* smooth muscle; *uLM,* longitudinal smooth muscles surrounding the urethra; *uCM,* circular smooth muscle surrounding the urethra; *Ur,* urethra; *V,* vagina; *Vm,* smooth muscle from the vaginal wall

The distribution of smooth and skeletal muscle tissues around the female urethra was identified using immunostaining with an anti-smooth muscle antibody and EVG staining to highlight the striated structure of muscle cells, respectively. From the 78 horizontal sections around the urethra, four representative levels, from proximal to distal, are shown (Figs. 1 and 2). In the upper urethra, the muscle layers were arranged from inner to outer as follows: longitudinal smooth muscle of the urethra (uLM), circular smooth muscle of the urethra (uCM), and skeletal muscle layers, which were identified as the EUS (Fig. 2a–c). Furthermore, the outermost smooth muscle tissue protruded anterolaterally and extended the muscle fibers toward the pubic bone (Fig. 2b–c, arrowheads). This finding was observed in two specimens which included the pubic bone. A 7 mm section distal to the previous section is shown in Fig. 2d–f. Muscle layers around the urethra were arranged in the same order. The skeletal muscle surrounded the urethra in a horseshoe shape and lacked muscle fibers posteriorly. In the urethra, the uCM and outermost smooth layers merged posteriorly (Fig. 2e, star). Sections 3 mm distal to Fig. 2d are shown in Fig. 2g–i. uLM and uCM extended anteriorly to separate the skeletal muscle layers (Fig. 2h and i, dagger). Figure 2j–l show sections 7 mm below the previous section. No skeletal muscle layers are observed at this level.

**Fig. 3:**
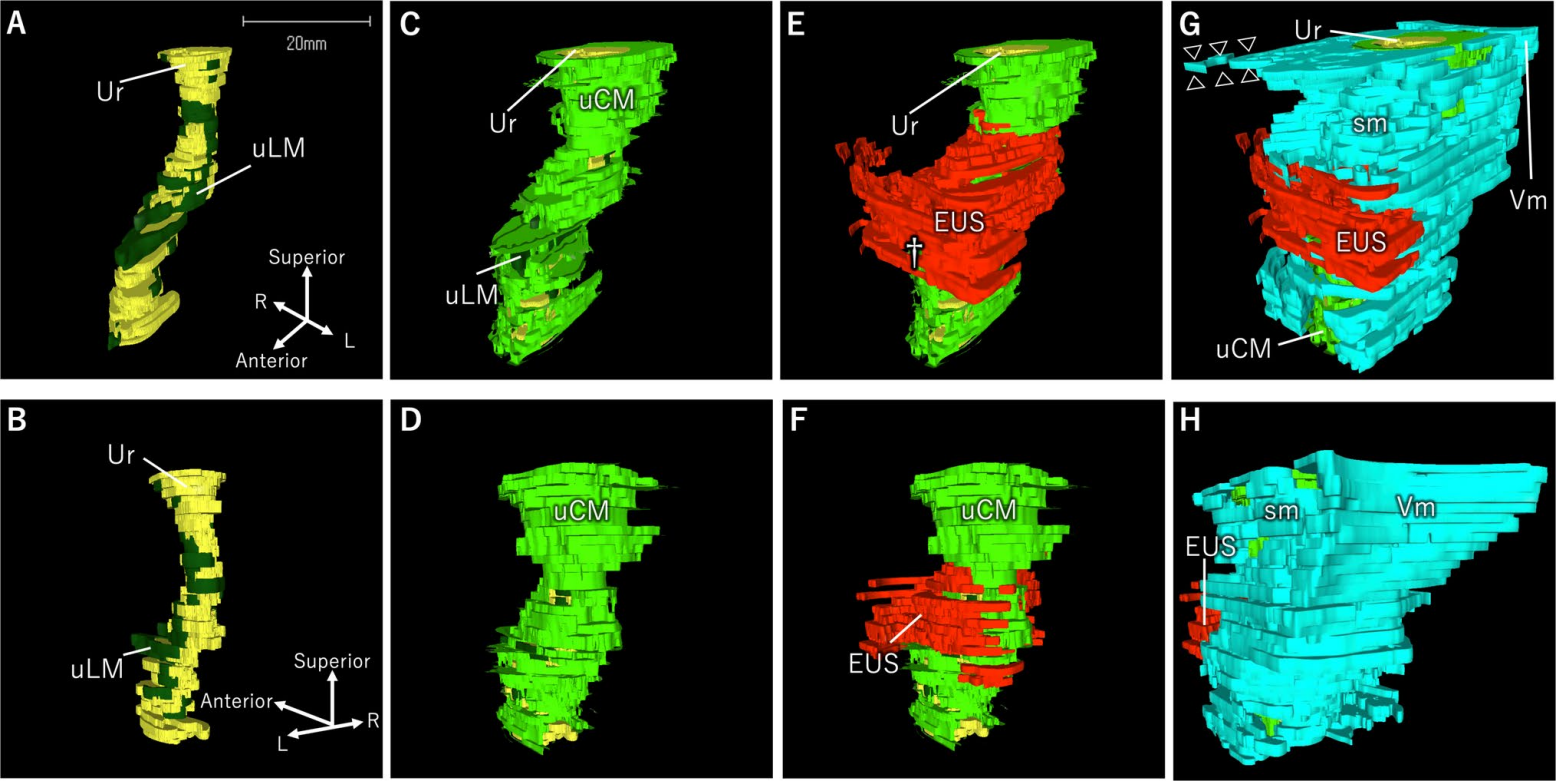
Three-dimensional reconstructed images of the muscular tissue around the urethra. Three-dimensional reconstructed images showing the muscles surrounding the female urethra. (a, c, e, and g) Anterior views. (b, d, f, and h) Posterior views. **a**, **b** The urethra was curved. The entire urethra was lined with uLM. **c**, **d** The entire length was covered by the cUM layer. **e**, **f** In the middle part of the urethra, skeletal muscle surrounds the ventral side of the urethra, which extends in a horseshoe shape and spreads side-to-side. Additionally, in the distal part of the urethra, uLM and uCM protruded anteriorly (e, dagger). **g**, **h** The smooth muscle layer covers the uLM, uCM, and EUS layers. The outermost smooth muscle layer was continuous with the smooth muscles of the vaginal wall. *EUS,* external urethral sphincter; *sm,* smooth muscle; *uLM,* longitudinal smooth muscle surrounding urethra; *uCM,* circular smooth muscle surrounding urethra; *Ur,* urethra; *Vm,* smooth muscle from the vaginal wall

Three-dimensional reconstructed images of the muscular tissue surrounding the female urethra were made according to the distribution of muscle tissue in the female urethra (Fig. 3). The urethra was curved, with the distal part curved forward along the vagina. The entire urethra was lined with uLM (Fig. 3a and b) and covered with a uCM layer (Fig. 3c and d). The middle part of the urethra was surrounded by skeletal muscle identified as the EUS (Fig. 3e); however, this muscle does not fully encircle the urethra, leaving the dorsal side uncovered (Fig. 3f). The ventral part of this muscle extends in a horseshoe shape and spreads side-to-side. A smooth muscle layer covers the uLM, uCM, and EUS (Fig. 3g and h) and is continuous with the smooth muscles of the vaginal wall. In the middle of the urethra, the smooth muscle layer is attached to the dorsal side of the bilateral expansion of the EUS.

### Muscle Tissue at the Lateral Side of the Female Urethra

**Fig. 4:**
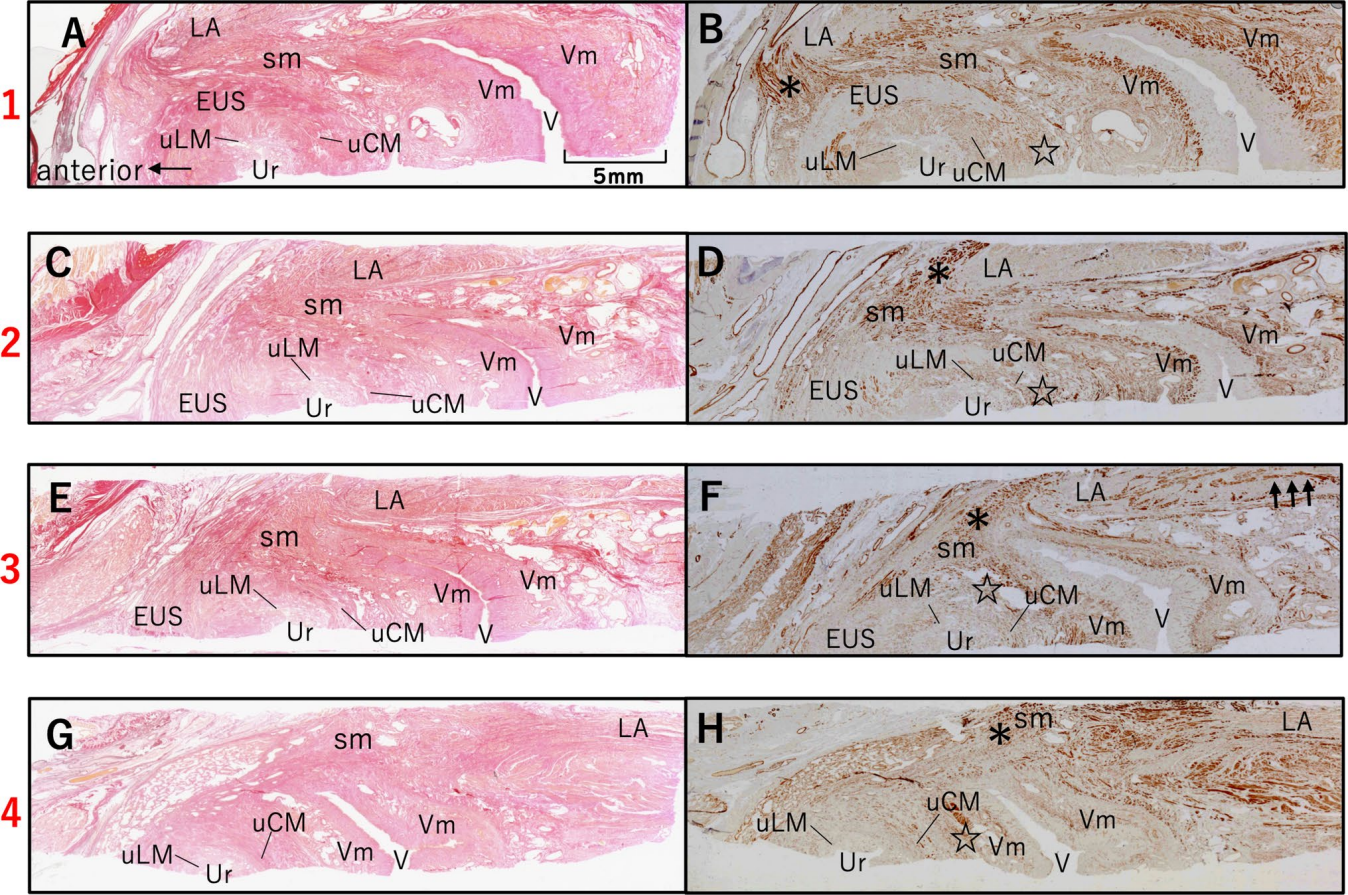
Histology of the smooth and skeletal muscular tissue of the lateral aspect of the urethra. Transverse histological sections of the tissue at the lateral aspect of the urethra using EVG staining (a, c, e, and g) and immunostaining with an anti-smooth muscle antibody (b, d, f, and h). **a**, **b** The upper urethra is arranged in the order of the uLM, uCM, and skeletal muscle from the inner to the outer layer. **c**, **d** A section 4 mm distal to the previous section. The skeletal muscle surrounded the urethra in a horseshoe shape and lacked muscle fibers posteriorly. Posteriorly, in the urethra, the uCM and outermost smooth layers continued with each other (d, star). **e**, **f** A section 2 mm distal to Fig. 3d. In the mid-urethra, smooth muscle fibers extending from the vagina were found between the LA and EUS (f *). Furthermore, these smooth muscle fibers were inserted into the LA (f, black arrows). **g**, **h** No skeletal muscle layer is observed in the lower urethra. *EUS,* external urethral sphincter; *LA,* levator ani; *sm,* smooth muscle; *uLM,* longitudinal smooth muscles surrounding the urethra; *uCM,* circular smooth muscle surrounding the urethra; *Ur,* urethra; *V,* vagina; *Vm,* smooth muscle from the vaginal wall

Specimens from the lateral side of the female urethra were collected to understand the relationship between EUS and LA. Of the 35 horizontal sections taken, four representative levels from proximal to distal are shown (Figs. 1 and 4). In the upper urethra, muscle layers were arranged from inner to outer: uLM, uCM, skeletal muscle identified as the EUS, and the outermost smooth muscle layer. This layer and the vaginal smooth muscle layer represent distinct muscular structures. However, no clearly defined boundary is observed (Fig. 4a and b). A section 4 mm distal to the previous section is shown in Fig. 4c and d, with muscle layers around the urethra arranged in the same order. The skeletal muscle surrounds the urethra in a horseshoe shape, lacking fibers posteriorly, where the uCM and outermost smooth layers merge (Fig. 4d, star). The section 2 mm distal to Fig. 4c is shown in Fig. 4e and f. In the mid-urethra, smooth muscle fibers extending from the vagina were found between the LA and EUS (Fig. 4f, *). Furthermore, these smooth muscle fibers were inserted into the LA (Fig. 4f, black arrows). Figure 4g and h show sections 6 mm below the previous section. No skeletal muscle layers are observed at this level.

## Discussion

This study clarified the distribution of muscle tissue around the urethra in women and the relationship between EUS and LA. Our research showed that four layers of muscle surround the female urethra: uLM, uCM, skeletal muscle (EUS), and the outermost smooth muscle layer from the inner to the outer layer. The smooth muscle layer was contiguous with the smooth muscles of the vaginal wall. Smooth muscle fibers extending from the vaginal wall were found between the LA and EUS. This finding indicates that the LA muscle influences urinary function by interacting with the smooth muscle between the LA and EUS.

Previous studies have described three muscle layers surrounding the female urethra: uLM, uCM, and striated muscle from the inner to the outer layer. The morphological characteristics of each muscle layer have been previously reported. For example, some studies reported that the uCM is thinner than the uLM, and the striated sphincter layer encircles the urethra, with the thickest muscle layer in the middle [4, 5]. However, its extension into the surrounding muscle layers has not been reported in detail. Our findings revealed four rather than three layers of muscle surrounding the female urethra. In addition to the three layers discussed in previous studies, there is an outermost smooth muscle layer. Previous studies have mainly focused on the sphincteric action of the internal urethral sphincter and EUS in relation to urethral control and contraction in women [5, 21]. Consequently, the EUS in women is widely regarded as a key component in controlling urethral contraction and managing urinary incontinence [22, 23]. Our study highlights the presence of the outermost smooth muscle layer and its connective role within the skeletal muscle groups of the female urethra and its essential role in urinary control.

This study showed that the urethra is surrounded by the outermost smooth muscle layer, which is contiguous with the smooth muscle of the vaginal wall. This smooth muscle is not only attached to the LA muscle, as reported by Kato et al. [4], but is also connected to the EUS. The smooth muscle may likely be involved in elevating the LA, thereby influencing the action of the EUS. When the LA contracts, it pulls this smooth muscle upward and forward, which subsequently supports the posterior and inferior aspects of the urethra. It is hypothesized that urethral fixation by smooth muscles enhances the contractile force of the EUS. The horseshoe-shaped structure of the EUS, which surrounds the anterior and lateral sides of the urethra, is believed to support this function.

Moreover, this smooth muscle layer protruded anterolaterally and extended its muscle fibers toward the pubic bone. In the proximal part of the female urethra, the smooth muscle extending toward the pubic bone is a part of the pubo-urethral ligament [4]. This structure further supports the idea that smooth muscles pull the urethra upward and forward, thereby stabilizing it and aiding the EUS in urethral closure. The anterior curvature of the distal urethra in some specimens may reflect this elevating action of the smooth muscle (Fig. 3a and b). In male individuals, the EUS is continuous with the external anal sphincter (EAS) and LA; therefore, the supportive action of the EAS and LA in EUS contractions is thought to contribute to its urinary function. In addition, smooth muscle tissue is present on the dorsal side of the urethra in male individuals and is believed to facilitate the contractile function of EUS [16]. However, in female individuals, EUS is not directly continuous with the LA or EAS; therefore, urinary function may depend significantly on the balance of the entire pelvic floor, particularly on urethral fixation by the smooth muscle. The uLM and uCM do not form a simple ring around the urethra; instead, these layers extend anteriorly, separating the skeletal muscle layer, which forms a horseshoe shape around the urethra without muscle fibers posteriorly and extends ventrally in a horseshoe shape, spreading side to side.

The relationship between urethral muscles and LA is still debated. In men, a study has shown continuity between the skeletal muscles of the EUS, LA, EAS, and bulbospongiosus. That study also revealed that the smooth muscles of the rectum fit into the spaces created by these muscles [15]. For women, some studies indicated that the LA may not actively participate in urethral control because it does not encircle the ventral side of the urethra [10]. In contrast, others reported that proper LA function is necessary for EUS function since the lower portion of the female EUS is connected to the puborectal portion of the LA by tendons [11]. A previous study by our research group on female urethra showed that vaginal smooth muscle fibers covered the urethra. We found that the LA and smooth muscle fibers were in direct contact and that the LA may have an impact on the function of the female urethra [4]. In addition, our current study clarified that smooth muscle fibers extend from the vagina between the LA and EUS in female individuals. Smooth muscle fibers were in direct contact with the LA and EUS, mediating the relationship between them. Thus, the LA may affect the function of the female urethra even without a direct connection to the urethral sphincter.

Understanding the distribution of the muscles around the urethra in women is clinically significant. To date, attention has primarily been focused on the urethral sphincter muscles when considering urinary control. However, our findings suggest that urinary function depends on the urethral sphincter and the balance with surrounding structures. The pelvic floor is susceptible to aging, and its structure descends with age [24, 25]. Under such conditions, it is necessary to consider both the urethral structure and function and the balance of the pelvic floor.

In addition to the sphincter effect, the striated urethral sphincter acts as a support structure to maintain stability and urinary continence [26]. The inner smooth muscle surrounding the urethra is thought to enhance the EUS force [25]. In particular, uLM contraction can shorten urethral length, lower closure pressure, and strengthen the sphincter mechanism, which is essential for initiating urination [5, 27]. This study revealed that the arrangement of the muscle layers differs depending on the urethral height, which may result in different effects on urination. The discovery that the urethra is not uniform and that its functional roles may vary depending on its height is crucial.

This study had some limitations. First, the cadaver donors were older adults with an average age of over 78 years, which could mean the weaker muscles in the aging female urethra may influence the muscle distribution. Second, the information on BMI, medical history, specifically obstetric history, vaginal deliveries, and history of pelvic/vaginal surgeries (prolapse/incontinence) of the cadavers was not possible because such information was not collected during the body donation process.

## Conclusions

This study revealed four muscle layers surrounding the female urethra and showed that the EUS is connected to the LA through the outermost smooth muscle layer. Therefore, increased LA activity can affect the urethra via this smooth muscle. Consequently, urination control involves not only the sphincteric action of the EUS but also the balance of the pelvic floor muscles.

## Data Availability

Data supporting this study’s findings are available from the corresponding author on a reasonable request.
